# Tracking Regional Tissue Volume and Function Change in Lung Using Image Registration

**DOI:** 10.1155/2012/956248

**Published:** 2012-10-18

**Authors:** Kunlin Cao, Gary E. Christensen, Kai Ding, Kaifang Du, Maghavan L. Raghavan, Ryan E. Amelon, Kimberly M. Baker, Eric A. Hoffman, Joseph M. Reinhardt

**Affiliations:** ^1^GE Global Research, Niskayuna, NY 12309, USA; ^2^Department of Electrical and Computer Engineering, The University of Iowa, Iowa City, IA 52242, USA; ^3^Department of Radiation Oncology, The University of Iowa, Iowa City, IA 52242, USA; ^4^Department of Radiation Oncology, Virginia Commonwealth University, Richmond, VA 23298, USA; ^5^Department of Biomedical Engineering, The University of Iowa, 1402A SC, Iowa City, IA 52242, USA; ^6^Department of Internal Medicine, The University of Iowa, Iowa City, IA 52242, USA

## Abstract

We have previously demonstrated the 24-hour redistribution and reabsorption of bronchoalveolar lavage (BAL) fluid delivered to the lung during a bronchoscopic procedure in normal volunteers. In this work we utilize image-matching procedures to correlate fluid redistribution and reabsorption to changes in regional lung function. Lung CT datasets from six human subjects were used in this study. Each subject was scanned at four time points before and after BAL procedure. Image registration was performed to align images at different time points and different inflation levels. The resulting dense displacement fields were utilized to track tissue volume changes and reveal deformation patterns of local parenchymal tissue quantitatively. The registration accuracy was assessed by measuring landmark matching errors, which were on the order of 1 mm. The results show that quantitative-assessed fluid volume agreed well with bronchoscopist-reported unretrieved BAL volume in the whole lungs (squared linear correlation coefficient was 0.81). The average difference of lung tissue volume at baseline and after 24 hours was around 2%, which indicates that BAL fluid in the lungs was almost absorbed after 24 hours. Regional lung-function changes correlated with the presence of BAL fluid, and regional function returned to baseline as the fluid was reabsorbed.

## 1. Introduction

 Bronchoalveolar lavage (BAL) has important clinical applications and is typically used to diagnose lung diseases, such as infection [1], lung cancer, and interstitial lung disease. During the BAL procedure, fluid is squirted into a small part of the lung through a bronchoscope and then recollected for examination.

It is of great interest to understand the progress of the distribution and resolution of BAL. Kelly et al. [2] used a digital subtraction technique to visualize the anatomical distribution of saline containing a low concentration of radio-opaque dye. Gurney et al. and Chen et al. [3, 4] showed that the extent and frequency of defects tended to decrease with time, and cleared after approximately 24 hours. Gabe et al. [5] observed fluid movement between lobes and between lungs before eventual resolution and demonstrated that lobes returned to their baseline after 24 hours. In addition, the change of lung function due to BAL procedure is important to study the effect of unretrieved BAL. Klein et al. [6] demonstrated that lung mechanics can be significantly altered an hour or longer after BAL. However, few studies have been done to track the resolution process of unretrieved BAL and quantify the BAL effects on lung ventilation function of a regional level.

Multidetector-row computed tomography (MDCT) can be used to acquire multiple static breath-hold CT images of the lung taken at different lung volumes. Applying image registration techniques to CT data, we are able to find dense deformation fields that transform the lungs between different lung volumes. The transformations can be analyzed to calculate voxel-by-voxel density change, estimate local lung tissue expansion, and make other biomechanical measurements. When combined with image segmentation results, functional and biomechanical measurements can be reported on a lung, lobe, and any arbitrarily shaped subregion basis [7].

Hoffman and Ritman [8] used CT density measurements to calculate regional tissue and air content of the lung. This method reflects tissue density accurately and estimates regional ventilation efficiently [5, 9]. Christensen et al. and Reinhardt et al. have estimated rates of local tissue deformation using a Jacobian-based ventilation measure [7, 10]. These measurements of tissue content and ventilation function can be utilized with image registration to track tissue volume change and mechanical property change regionally over time.

This paper describes an image registration based method to quantitatively track the resolution process of unretrieved BAL and measure regional lung ventilation function during 24 hours after BAL procedure. We evaluate our registration by tracking landmark movements. We show that the unretrieved BAL is gradually absorbed and the non-air content returns to the baseline after 24 hours. In addition, we observed the local tissue ventilation function returns to baseline state after 24 hours.

This study is most similar to [5]. They both observed the resolution progress after BAL. The main difference is that in [5] the BAL resolution progress was studied at the lobe level which needs the lobe segmentation for each dataset, while this paper presents a method to study the BAL resolution at a regional level utilizing image registration techniques. Also, local lung function change due to BAL was observed in this work.

## 2. Material and Methods

### 2.1. Image Data Sets

**Figure 1:**
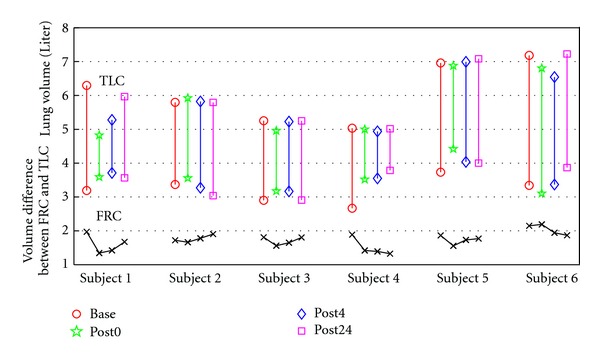
Lung volumes at FRC and TLC pressures, and the volume difference between FRC and TLC scans in each phase for six subjects.

The protocol was reviewed and approved by the University of Iowa Institutional Review Board. CT data sets from six healthy human subjects were used. Each subject was scanned at four time points: baseline, immediate post-lavage (within 30 minutes), 4 hours post-lavage, and 24 hours post-lavage. At each time point a pair of Functional Residual Capacity (FRC) and Total Lung Capacity (TLC) scans were acquired. Therefore, each subject experienced totally eight CT scans from four phases during a 24-hour period. Each scan pair was acquired with a Siemens Sensation 64 multi-detector row CT scanner (Forchheim, Germany) during breath-holds in the same scanning phase. Each volumetric data set was acquired at a section spacing of 0.5 mm and a reconstruction matrix of 512 × 512. In-plane pixel spacing was approximately 0.6 mm × 0.6 mm. Scans were reconstructed using a B31f reconstruction kernel.  Table 1 lists the data sets acquired for each subject in four phases and the name of each scan used in this paper.  Figure 1 shows the lung volumes at FRC and TLC pressures, and the volume difference between FRC and TLC scans in each phase for six subjects.

Subjects were lavaged in the right middle lobe and ligula. Each subject received aliquots of 300 mL in total; the total amount is denoted as *V*_BAL_total__. BAL fluid was also retrieved and measured by the bronchoscopist during this procedure; the retrieved amount is denoted as *V*_BAL_retrieved__. Then the subjects underwent the post-lavage scan within 30 minutes of lavage (post0 phase scans). The volume of unretrieved BAL fluid *V*_BAL_ can be calculated by subtracting the volume retrieved from the volume delivered, as shown in (1). The calculated *V*_BAL_ for each subject is shown in Table 2. Note that subject 2 was observed to cough a significant amount of BAL fluid out of the lungs.

(1) $$\begin{array}{c} V_{\text{BAL}}=V_{{\text{BAL}}_{\text{total}}}-V_{{\text{BAL}}_{\text{retrieved}}}. \end{array}$$

### 2.2. Method Overview

**Figure 2:**
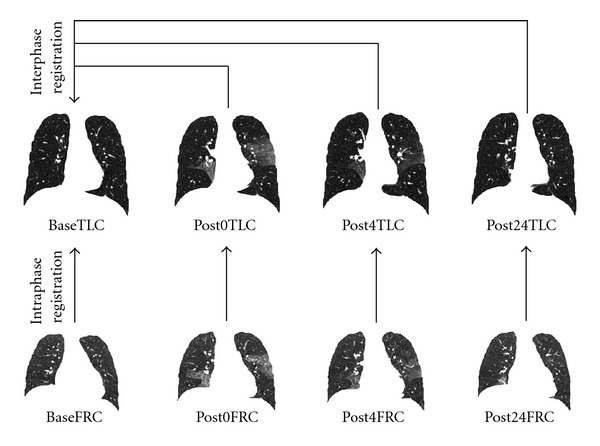
For each subject, intraphase registrations register the FRC image to the TLC image within a phase; interphase registrations register all TLC images in post-lavage phases to baseline TLC.

 Our goal is to utilize image registration to track non-air volume change and tissue ventilation change regionally during 24 hours after BAL procedure.  Figure 2 shows the registrations used for analysis. Two types of registration were performed on the CT data sets. Intra-phase registrations register the FRC image to the TLC image within a phase. These results are used to estimate local lung function in each phase and make the comparison between different phases to measure the function change. Inter-phase registrations register all TLC images in post-lavage phases to the baseline TLC. These results are used to track local tissue (or non-air) content change across four different phases.

### 2.3. Tissue Volume Assessment

 We assume that lung is a mixture of air and tissue/blood (non-air). So the Hounsfield units (HU) in lung CT images are a function of tissue and air content. From the HU of CT lung images, the tissue volume and air volume can be estimate following the air-tissue mixture model by Hoffman and Ritman [8]. The tissue volume *V* in a voxel at position **x** can be estimated as

(2) $$\begin{array}{c} V\left(x\right)=v\left(x\right)\frac{\text{HU}\left(x\right)-\text{H}{\text{U}}_{\text{air}}}{\text{H}{\text{U}}_{\text{tissue}}-\text{H}{\text{U}}_{\text{air}}}=v\left(x\right)\beta \left(I\left(x\right)\right), \end{array}$$

where *v*(**x**) is the volume of voxel **x**. Similarly, the air volume *V*′ in a voxel can be estimated as

(3) $$\begin{array}{c} V\prime \left(x\right)=v\left(x\right)\frac{\text{H}{\text{U}}_{\text{tissue}}-\text{HU}\left(x\right)}{\text{H}{\text{U}}_{\text{tissue}}-\text{H}{\text{U}}_{\text{air}}}=v\left(x\right)\alpha \left(I\left(x\right)\right). \end{array}$$

In this paper, we assume that HU_air_ = −1000 and HU_tissue_ = 55. *α*(*I*(**x**)) and *β*(*I*(**x**)) are introduced for notational simplicity, and *α*(*I*(**x**)) + *β*(*I*(**x**)) = 1. The tissue volume in a region are calculated as

(4) $$\begin{array}{c} \text{TV}=\int_{R}V\left(x\right)dx=\int_{R}v\left(x\right)\beta \left(I\left(x\right)\right)dx. \end{array}$$

### 2.4. Image Registration

 The goal of registration is to find the spatial mapping that brings two images into alignment. Let *I*_1_ and *I*_2_ represent two 3D image volumes to be registered. The transform defines how points from the template image *I*_1_ are mapping to their corresponding points in the target image *I*_2_. In three dimensional space, let **x** = (*x*_1_, *x*_2_, *x*_3_)^*T*^ define a voxel coordinate in the image domain. The transformation **h** is a (3 × 1) vector-valued function defined on the voxel lattice of target image, and **h**(**x**) gives its corresponding location in template image.

For each subject, two different kinds of registrations were performed: intra-phase registration matches a FRC data to its according TLC data in the same phase and inter-phase registration matches the TLC data in post-lavage phases to the baseline TLC data. Different registration algorithms were used to perform intra-phase and inter-phase registrations.

#### 2.4.1. Intra-Phase Registration: RTVP

 A regularized tissue volume and vesselness measure preserving nonrigid registration (RTVP) algorithm [11, 12] was used to estimate the transformations from FRC to TLC in the same phase. The RTVP algorithm minimizes the sum of squared tissue volume difference (SSTVD) [9, 13, 14] and vesselness measure difference (SSVMD), utilizing the rich intensity and shape information provided by the vessels. This method has been shown to be effective at registering across lung CT images with high accuracy [11, 12].

For a pair of FRC and TLC scans in the same phase, the time interval of acquisition can be ignored and the tissue volume is assumed unchanged. Therefore, the registration can be driven by preserving tissue volume in two images. The sum of squared tissue volume difference (SSTVD) similarity cost function [9] accounts for the variation of intensity in the lung CT images during respiration. This similarity criterion minimizes the local difference of tissue volume inside the lungs scanned at different pressure levels. The tissue volume of a CT scan can be estimated by (2). Then the intensity similarity metric SSTVD is defined as [9]

(5) CSSTVD=∫Ω[V2(x)−V1(h(x))]2dx=∫Ω[v2(x)β(I2(x))−v1(h(x))β(I1(h(x)))]2dx,

where *I*_1_ and *I*_2_ are the template and target image intensity functions, respectively. *Ω* denotes the union of lung regions in target image and deformed template image. The Jacobian of a transformation *J*(**h**) estimates the local volume changes resulted from mapping an image through the deformation. Thus, the tissue volumes in image *I*_1_ and *I*_2_ are related by *v*_1_(**h**(**x**)) = *v*_2_(**x**) · *J*(**h**(**x**)).

As the blood vessels branch to smaller diameters, the raw grayscale information from vessel voxels provide very little contribution to guide the intensity-based similarity metrics. In order to better utilize the information of blood vessel locations, we use the vesselness measure based on the eigenvalues of the Hessian matrix of image intensity. Ordering the eigenvalues of a Hessian matrix by magnitude |*λ*_1_| ≤ |*λ*_2_| ≤ |*λ*_3_|, Frangi's vesselness function [15] is defined as

(6) F(λ)={(1−e−RA2/2α2)·e−RB2/2β2 ·(1−e−S2/2γ2),if  λ2<0  and  λ3<0,0,otherwise,

with

(7) $$\begin{array}{c} R_{A}=\frac{\left|\lambda_{2}\right|}{\left|\lambda_{3}\right|},\;\;R_{B}=\frac{\left|\lambda_{1}\right|}{\sqrt{\left|\lambda_{2}\lambda_{3}\right|}},\;\;S=\sqrt{\lambda_{1}^{2}+\lambda_{2}^{2}+\lambda_{3}^{2}}, \end{array}$$

where *R*_*A*_ distinguishes between plate-like and tubular structures, *R*_*B*_ accounts for the deviation from a blob-like structure, and *S* differentiates between tubular structure and noise. *α*, *β*, and *γ* control the sensitivity of the vesselness measure. The experiments in this paper used *α* = 0.5, *β* = 0.5, and *γ* = 5. The vesselness measure is calculated in multiscales and selected as the maximum response. The vesselness image is rescaled to (0, 1) and can be considered as a probability-like estimate of vesselness features. The feature-based similarity metric, sum of squared vesselness measure difference (SSVMD), is designed to match similar vesselness patterns in two images. Given *F*_1_(**x**) and *F*_2_(**x**) as the vesselness measures of images *I*_1_ and *I*_2_ at location **x** respectively, this new cost function is formed as

(8) $$\begin{array}{c} C_{\text{SSVMD}}=\int_{\Omega }{\left[F_{2}\left(x\right)-F_{1}\left(h\left(x\right)\right)\right]}^{2}. \end{array}$$

Enforcing constraints on the transformation helps generate physiologically more meaningful registration results. Continuum mechanical models such as linear elasticity can be used to regularize the transformations. In this paper, a Laplacian operator is used to regularize the displacement fields **u** where **u** = **h**(**x**) − **x**. This regularization term is formed as

(9) $$\begin{array}{c} C_{\text{LAP}}=\int_{\Omega }{||\nabla^{2}u(x)||}^{2}dx, \end{array}$$

where ∇ = [∂/∂*x*_1_, ∂/∂*x*_2_, ∂/∂*x*_3_] and ∇^2^ = ∇·∇ = [(∂^2^/∂*x*_1_^2^) + (∂^2^/∂*x*_2_^2^)+(∂^2^/∂*x*_3_^2^)]. Using linear elasticity differential operator can help smooth the transformation, and help eliminate abrupt changes in the displacement fields.

Finally, the total cost is defined as a linear combination of the intensity-based metric, vesselness measure preserving metric and Laplacian constraint

(10) $$\begin{array}{c} C_{\text{TOTAL}}=C_{\text{SSTVD}}+\chi C_{\text{SSVMD}}+\gamma C_{\text{LAP}}. \end{array}$$

Constants *χ* and *γ* are weights to adjust the significance of the three terms. In this work, the weighting constants were selected by minimizing three separate cost terms at the same time based on the registration experiments of data sets from six subjects. These parameters were set as *χ* = 1 and *γ* = 0.01 for all intra-phase RTVP registrations.

The transformation **h**(**x**) was parameterized using a cubic B-splines represented transform:

(11) $$\begin{array}{c} h\left(x\right)=x+\sum_{i\in G}\phi_{i}\beta \left(x-x_{i}\right), \end{array}$$

where *ϕ*_*i*_ describes the displacements of the control nodes and *β*(**x**) is a three-dimensional tensor product of basis functions of cubic B-Spline. A spatial multiresolution procedure from coarse to fine was used in the registration to improve speed, accuracy and robustness. The total cost in (10) was optimized using a limited-memory, quasi-Newton minimization method with bounds (L-BFGS-B) [16] algorithm. Based on the sufficient conditions to guarantee the local injectivity of functions parameterized by uniform cubic B-Splines proposed by Choi and Lee [17], the B-Splines coefficients are constrained so that the transformation maintains the topology of two images.

#### 2.4.2. Inter-Phase Registration: RIVP

 Since the effect of the BAL fluid resolved within 24 hours after lavage, the tissue volume (non-air volume) varied between different phases. Therefore, the tissue volume preserving assumption is not valid in the case when registering TLC scans from post-lavage phases to baseline TLC scan. The sum of squared difference (SSD) defined by

(12) $$\begin{array}{c} C_{\text{SSD}}=\int_{\Omega }{\left[I_{2}\left(x\right)-I_{1}\left(h\left(x\right)\right)\right]}^{2}dx, \end{array}$$

which was used for inter-phase registration. The underlying assumption of SSD is that the image intensity at corresponding points between two images should be similar. This is true when registering images of the same modality. In such cases, if the images are perfectly mapped, the corresponding intensities should be identical, which means each point of the same underlying structure has the same intensity value in the two images to be registered. However, considering the change in CT intensity due to different air content and BAL effects, the grayscale ranges were different within the lung region in two TLC images acquired at different phases. To balance these grayscale range differences, normalization of the intensities is needed. A histogram matching procedure was used before SSD driven registration to modify the histogram of template image so that it was similar to that of target image.

Except for the fact that the similarity metric is changed from SSTVD to SSD (after histogram matching), other components and registration schemes of inter-phase registration are the same with those of intra-phase registration. The inter-phase TLC scans matching uses a regularized intensity and vesselness preserving nonrigid registration (RIVP). The transformation is estimated by minimizing the sum of squared intensity difference, the sum of squared vesselness measure difference, and the Laplacian constraints. The total cost function

(13) $$\begin{array}{c} C_{\text{TOTAL}}=C_{\text{SSD}}+\chi C_{\text{SSVMD}}+\gamma C_{\text{LAP}}. \end{array}$$

In this paper, the weighting constants were chosen using similar criteria to that in intra-phase RTVP registrations, and were set as *χ* = 2 and *γ* = 10 for all inter-phase RIVP registrations.

### 2.5. Assessment of Registration Accuracy by Tracking Landmark Movement

**Figure 3:**
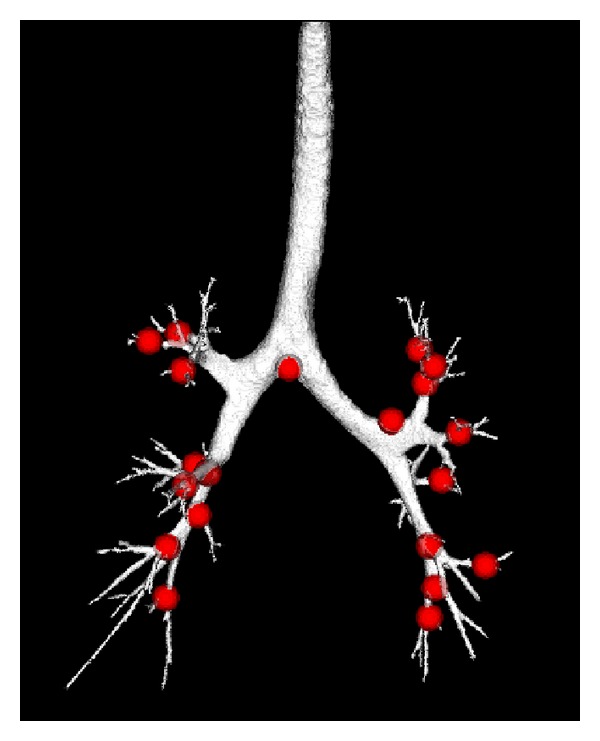
Distribution of landmark positions (red points) selected on the airway tree from the baseTLC image of one subject.

 For each subject, 20 anatomic landmarks were manually selected and tracked in all eight images during four phases to assess registration accuracy. These landmarks were chosen as recognizable airway branchpoints, as shown in Figure 3. The transformation determined from the lung registration can be used to predict the landmark movement between the different coordinates. Landmark error is defined as the Euclidean distance between registration-predicted landmark position and its true position in the same image coordinate to measure the matching accuracy.

**Figure 4:**
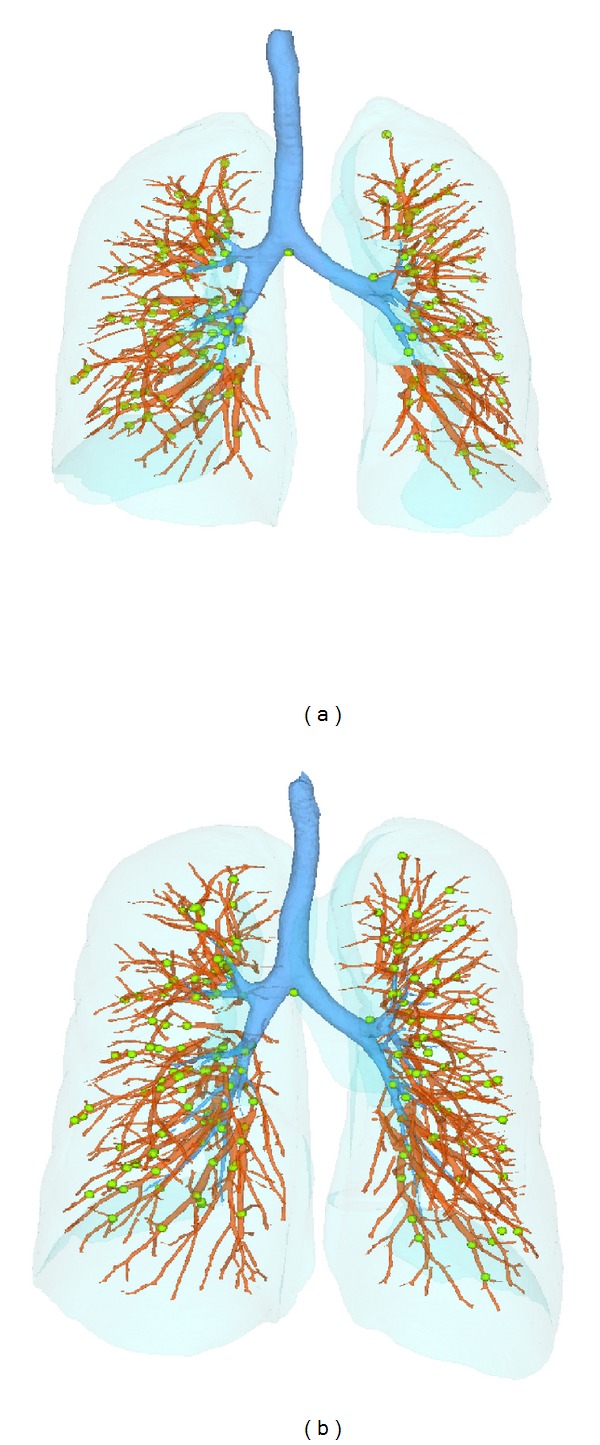
Distribution of landmarks (green points) selected at vessel-tree branch points on (a) FRC, and (b) TLC scans of one subject.

 For each scan pair of baseline data, a distributed set of landmarks selected as vessel tree branch points were also defined. The landmarks in baseFRC image were first selected as the bifurcations of the segmented vessel tree [18]. A semi-automatic system [19] was used to guide the observer to find the landmarks in the baseTLC image with their corresponding voxels in the baseFRC image. The landmarks were selected throughout the lungs. An example of the point distribution is shown in Figure 4. An expert selected over 100 landmark pairs for each baseline scan pair of the six subjects. 

### 2.6. Tracking Lung Tissue Volume Change

 For each subject, intersection registrations mapped all the post-lavage TLC images to baseline coordinate system, and provided voxel-wise correspondences from the baseline phase to the three post-lavage phases. These mappings enable assessment of the tissue volume change for a given voxel position across the four phases.

**Figure 5:**
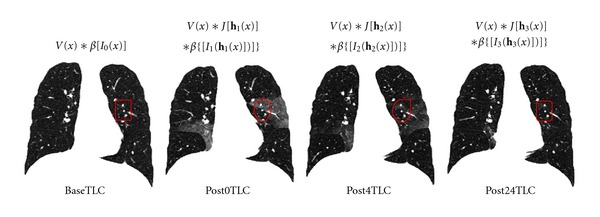
Illustration of the method utilizing inter-phase registration results to track tissue volume across four different phases. The region within the red rectangular in baseTLC is assumed to deform to the regions enclosed by red curves in images of post phases.

Using a Lagrangian reference frame, Figure 5 shows an example of a region at location **x** of baseTLC deforms to different shapes in post0TLC, post4TLC, and post24TLC under transformations **h**_1_, **h**_2_, and **h**_3_, respectively. Assume the region within the red rectangular in baseTLC corresponds to the regions enclosed by red curves in images of post phases in Figure 5. The volumes of the same region in four phases are *v*(**x**), *v*(**x**)*J*(**h**_1_(**x**)), *v*(**x**)*J*(**h**_2_(**x**)), and *v*(**x**)*J*(**h**_3_(**x**)). These volumes can be decomposed into the tissue volume fraction and air volume fraction based on the mean voxel intensity within the cube. The tissue volumes are calculated as *v*(**x**)*β*(*I*_0_(**x**)), *v*(**x**)*J*(**h**_1_(**x**))*β*(*I*_1_(**h**(**x**)), *v*(**x**)*J*(**h**_2_(**x**))*β*(*I*_2_(**h**(**x**)) and *v*(**x**)*J*(**h**_3_(**x**))*β*(*I*_3_(**h**(**x**)), respectively. Here *J* is denoted as the Jacobian of the transformations; *I*_0_, *I*_1_, *I*_2_, *I*_3_ are intensity function of the four images. As the ratio of tissue to air increases, the CT intensity of a voxel increases (getting brighter). In this way, we are able to track tissue volume of any subregion across the four phases using inter-phase registration results. 

 The total tissue volume in a region can be integrated using (4). In the same region *R* defined on baseline coordinate. Let TV_0_, TV_1_, TV_2_, and TV_3_ represent the total tissue volume from baseTLC, post0TLC, post4TLC, and post24TLC, respectively. They are calculated as

(14) $$\begin{array}{c} \text{T}{\text{V}}_{0}=\int_{R}v\left(x\right)\beta \left(I_{0}\left(x\right)\right)dx,\\ \text{T}{\text{V}}_{i}=\int_{R}v\left(x\right)J\left(h_{i}\left(x\right)\right)\beta \left(I_{i}\left(h_{i}\left(x\right)\right)\right)dx,\;i=1,2,3. \end{array}$$

Meanwhile, we define TVC_1_, TVC_2_, and TVC_3_ as the index of tissue volume change for three post-lavage phases when compared with base phase, and also define TVCR_1_, TVCR_2_ and TVCR_3_ as the index of tissue volume change ratio. They are formulated as follows:

(15) TVCi=TVi−TV0,  TVCRi=TVi−TV0TV0×100%,        i=1,2,3.

### 2.7. Assessment of Lung Function by Jacobian

 The lung tissue deformation pattern is an index to assess lung function. In this work, the Jacobian determinant of the transformation field derived by image registration is used to estimate the local tissue deformation [7].

The Jacobian determinant (often simply called the Jacobian) [20–22] is a measurement to estimate the pointwise expansion and contraction during the deformation. In three-dimensional space, let **h**(**x**) = [*h*_1_(**x**), *h*_2_(**x**), *h*_3_(**x**)]^*T*^ be the vector-valued transformation which deforms the template image *I*_1_ to the target image *I*_2_. The Jacobian of the transformation *J*(**h**(**x**)) at location **x** = (*x*_1_, *x*_2_, *x*_3_)^*T*^ is defined as

(16) $$\begin{array}{c} J\left(h\left(x\right)\right)=\left|\begin{array}{ccc} \frac{\partial h_{1}\left(x\right)}{\partial x_{1}} & \frac{\partial h_{1}\left(x\right)}{\partial x_{2}} & \frac{\partial h_{1}\left(x\right)}{\partial x_{3}}\\ \frac{\partial h_{2}\left(x\right)}{\partial x_{1}} & \frac{\partial h_{2}\left(x\right)}{\partial x_{2}} & \frac{\partial h_{2}\left(x\right)}{\partial x_{3}}\\ \frac{\partial h_{3}\left(x\right)}{\partial x_{1}} & \frac{\partial h_{3}\left(x\right)}{\partial x_{2}} & \frac{\partial h_{3}\left(x\right)}{\partial x_{3}} \end{array}\right|. \end{array}$$

Using a Lagrangian reference frame, a Jacobian value of one corresponds to zero expansion or contraction. Local tissue expansion corresponds to a Jacobian greater than one and local tissue contraction corresponds to a Jacobian less than one.

Across different phases, the Jacobian estimates are related to two factors. The first factor is the unretrieved BAL. According to [6], the lung mechanics can be significantly altered an hour or longer after BAL. The second factor is breathing effort. As shown in Figure 1, saline fluid retained in the lung after BAL procedure may cause reduced breathing effort, that is, smaller volume change from FRC to TLC. Smaller volume change results in lower lung function on average. In order to focus our analysis on how the unretrieved BAL affects the lung function, we eliminate the breathing effort factors by calculating the cumulative distribution function (CDF) of the Jacobian estimates.

In our case, CDF of Jacobian describes the probability that Jacobian values will be found less than or equal to a given value. Intuitively, it is the “area under curve” function of the probability distribution. It can be viewed as “rank” information: a CDF of 1 means rank top (largest Jacobian), while a CDF value of 0 means rank bottom (smallest Jacobian). In the following description, we call the CDF of the Jacobian as the rank for convenience. Regardless of different overall volume changes, regions with higher lung function correspond to higher rank. In this way, we eliminate the breathing effort factors while comparing lung function changes caused by unretrieved BAL fluid. Let Rank_0_, Rank_1_, Rank_2_, and Rank_3_ represent the rank of Jacobians estimated from base, post0, post4, and post24 phases, respectively. Then the rank change RC is defined as

(17) $$\begin{array}{c} \text{R}{\text{C}}_{i}=\text{Ran}{\text{k}}_{i}-\text{Ran}{\text{k}}_{0},\;i=1,2,3. \end{array}$$

### 2.8. Preprocessing

 Preprocessing starts by identifying the lung regions in all images using the Pulmonary Workstation 2.0 (VIDA Diagnostics, Inc., Iowa City, IA). For the baseTLC image of each subject, an automatic lobe segmentation algorithm [23] was used to segment the parenchyma regions into five different lobes. Images and masks are downsampled by a factor of 2 in each dimension to reduce computation time of image registration.

After preprocessing, three inter-phase registrations mapping TLC images from three post-lavage phases to baseline TLC image for each subject were performed using RIVP registration. These resulting transformations are used to track tissue volume change across four phases. Then four intra-phase registrations mapping FRC image to TLC image within each phase for each subject were performed using RTVP registration. The resulting transformation were used to estimate regional lung deformation pattern within each phase. Combined with inter-phase transformations, the regional lung function was tracked across four phases.

For the RTVP and RIVP registration, a multiresolution strategy was used in the optimization process. It proceeds from low to high image resolution starting at one-eighth the spatial resolution and increases by a factor of two until the full resolution is reached. Meanwhile, a hierarchy of B-spline grid spaces from large to small is used. The finest B-spline grid space used in the experiments is 8 mm. The images and image grid space were refined alternatively.

## 3. Results

### 3.1. Assessment of Registration Accuracy

**Figure 6:**
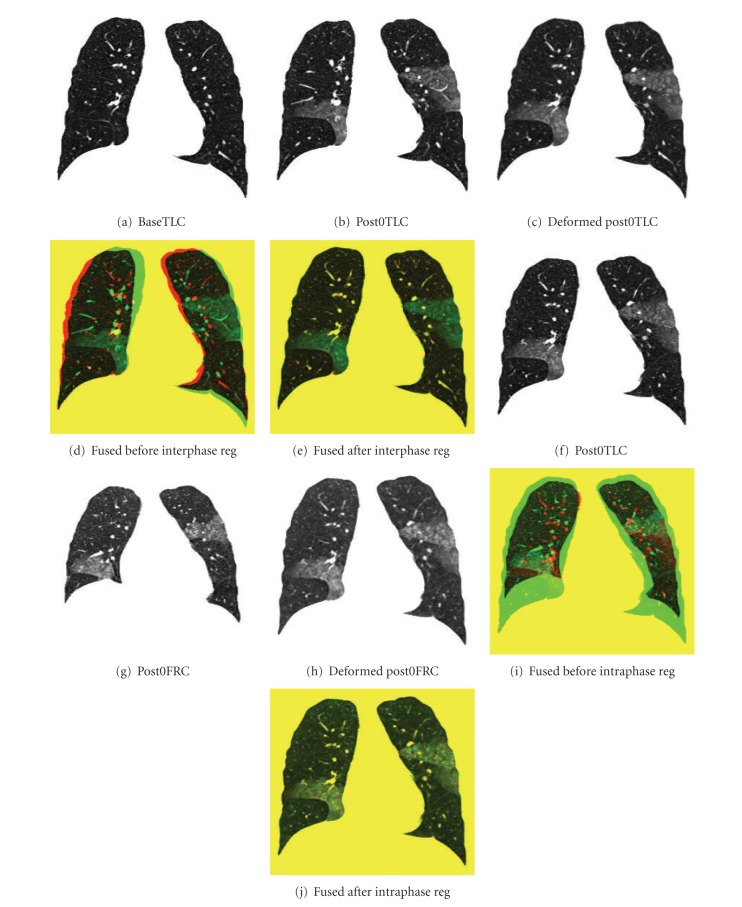
Example of registration results. Matching results of interphase registration: (a) a baseTLC slice; (b) a post0TLC slice; (c) the slice of deformed image from post0TLC to baseTLC which matches (a); (d) the fused slice of (a) (colored red) and (b) (colored green) before registration; (e) the fused slice of (a) and (c) after registration. Matching results of intraphase registration are shown in the same way in (f)–(j).

 Registration outcome can be judged qualitatively by observers through visual assessment. Visualization of the image intensity matching is an intuitive method to determine how well the region boundaries and corresponding structures were aligned. Examples of inter-phase and intra-phase registration results are shown in Figure 6. Figures 6(a)–6(e) show the matching results of inter-phase registration: (a) and (b) show coronal slices of baseTLC and post0TLC data sets for the same subject, respectively; the deformed slice from FRC image to TLC image is shown in (c); (d) shows the color-coded fused slice between (a) and (b) before registration; and (e) shows the color-coded fused slice between (a) and (c) after registration. Matching results of intra-phase registration between baseTLC and baseFRC are shown in the same way in Figures 6(f)–6(j). It is obvious to see that besides the lung boundaries, important structures, such as airway, vessel, and fissure locations, are aligned well after registration. 

**Figure 7:**
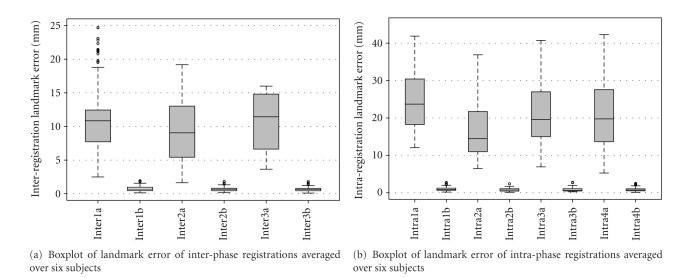
Registration accuracy on airway landmarks. (a) Boxplot of landmark error of interphase registrations. Inter1a, Inter2a and Inter3a denote the error from post0TLC, post4TLC, and post24 TLC to baseTLC before registration, respectively. While Inter1b, Inter2b and Inter3b denote the error after three interphase registrations. (b) Boxplot of landmark error of intraphase registrations. Intra1a, Intra2a, Intra3a, and Intra4a denote the error from baseFRC to baseTLC, from post0FRC to post0TLC, from post4FRC to post4TLC, and from post24FRC to post24TLC before registration, respectively. While Intra1b, Intra2b, Intra3b and Inter4b denote the error after four intraphase registrations.

**Figure 8:**
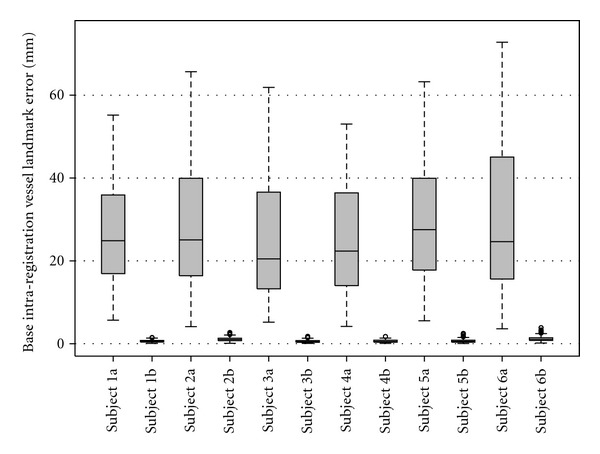
Registration accuracy on vessel landmarks of baseline intra-phase registration. For each subject, the left bar shows error before registration, and the right bar shows error after registration.

For each subject, registration accuracy is quantitatively assessed by tracking movement of 20 airway-branch landmarks on TLC images across four phases, and between FRC and TLC images within the same phase. Boxplots of the landmark tracking error of both inter-phase registration and intra-phase registration over six subjects are shown in Figure 7. The boxplot of landmark error on over 100 widely distributed vessel-branch landmarks for baseline intra-phase registration (warping baseFRC to baseTLC) is shown in Figure 8. These mean errors for each subject were on the order of 1 mm.

### 3.2. Comparison between Quantitative-Assessed and Bronchoscopist-Reported Unretrieved BAL

 According to the experimental protocol, the quantitative assessment of tissue volume change between baseline phase and post0 phase should be correlated with unretrieved BAL fluid from bronchoscopist reported data [5]. From (1), *V*_BAL_ is calculated from bronchoscopist-reported volumes instilled and retrieved during BAL procedure. From (15), the tissue volume change calculated between baseTLC and deformed post0TLC to baseline coordinate is defined as TVC_1_. Experiments to find the relationship of the quantitative assessment and clinical data were performed. The correlation coefficients between *V*_BAL_ and TVC_1_ were calculated by linear regression.

**Figure 9:**
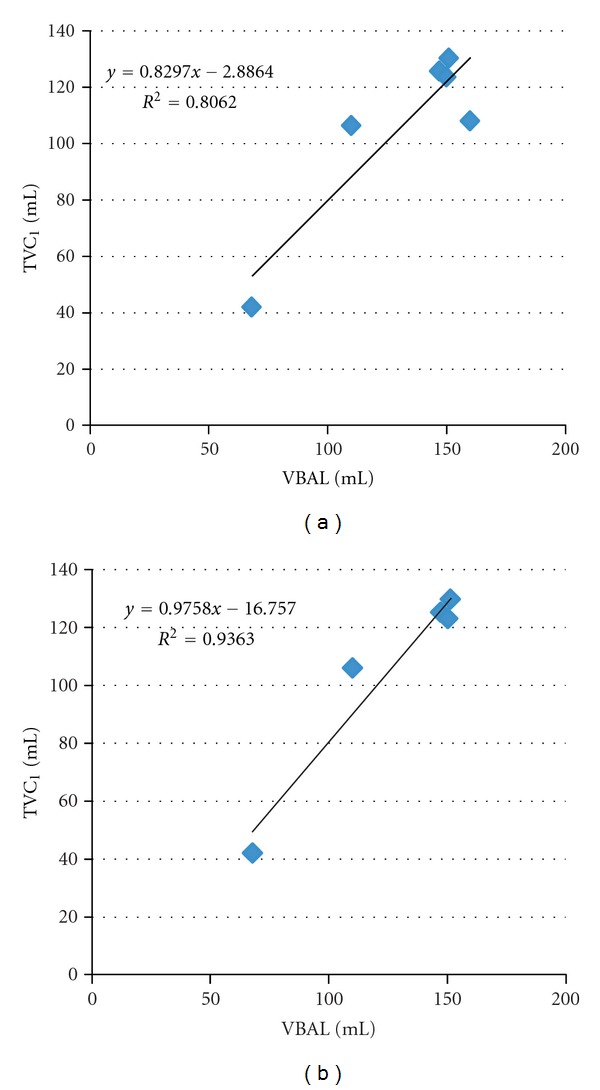
Correlation between quantitative assessment of TVC_1_ and bronchoscopist reported unretrieved BAL fluid volume *V*_BAL_. (a) shows the linear regression using data from all six subjects. (b) shows the linear regression using data from five subjects after removing subject 2.

Table 2 lists the volume of unretrieved BAL fluid *V*_BAL_ and tissue volume change TVC_1_ for each subject. The linear regression line and correlation coefficient are shown in Figure 9(a). As noted, subject 2 was observed to cough a significant amount of BAL fluid out of the lungs, which was not collected during lavage process. Therefore it was reasonable to remove this subject from the linear regression analysis. The linear regression line and correlation coefficient after removing data from subject 2 is shown in Figure 9(b).

### 3.3. Tracking Global and Regional Tissue Volume Change

 Using ([Disp-formula EEq14]), we calculated the tissue volume in the same arbitrary-shaped region *R* (defined on baseline phase) at different phases. Substituting *R* with the baseline whole lung segmentation, we tracked the global tissue volume change using (15). The measurements of the whole lung tissue volume change ratio over different phases are listed in Table 3. 

**Figure 10:**
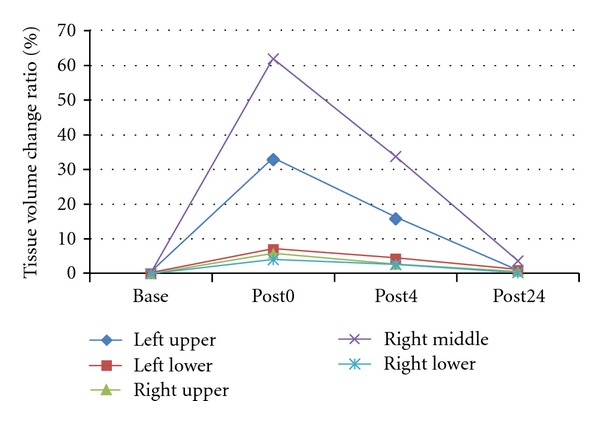
Tissue volume change ratio over different phases for five lobes average across six subjects.

For each scan pair of baseline data, an automatic lobe segmentation algorithm [23] was used to segment the parenchyma regions into five different lobes. Substituting *R* in ([Disp-formula EEq14]) with five different lobe segmentations, we tracked the lobe-based tissue volume change over time using (15).  Figure 10 shows the lobe-based tissue volume change ratio over different phases averaged across six subjects. 

### 3.4. Tracking Regional Lung Function Change

**Figure 11:**
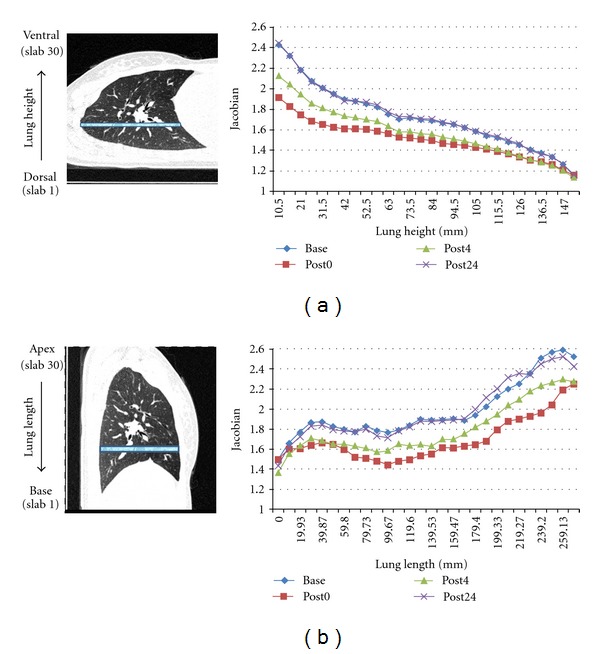
Lung expansion pattern within each phase for one subject. (a) shows the slab division scheme from dorsal to ventral lung, and the corresponding mean Jacobian in each slab over four phases. (b) shows the slab division scheme from apex to base lung, and the corresponding mean Jacobian in each slab over four phases.

 For each intra-phase registration, we calculated voxel-wise Jacobian from the transformations to estimate lung expansion and contraction, which reflects the local lung function during the respiration process. In order to observe the regional lung function tendency, we divided the lung into 30 rectangular-shaped slabs from dorsal to ventral lung, and from apex to base lung. The average Jacobian was calculated within each slab, and plotted in Figure 11. Each color-coded line shows the Jacobian estimated from one intra-phase registration. 

**Figure 12:**
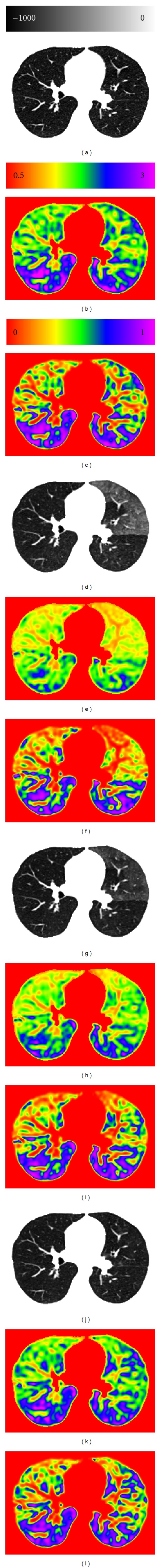
Comparison of local lung function estimates over different phases. (a), (d), (g), and (j) show the intensity pattern on the same slice from baseTLC, deformed post0TLC to baseTLC, deformed post4TLC to baseTLC, and deformed post24TLC to baseTLC, respectively. (b), (e), (h), and (k) show the Jacobian maps estimated from four phases on the slice. (c), (f), (i), and (l) show the corresponding rank maps of Jacobian estimates.

Combining intra-phase and inter-phase registration together, we mapped Jacobian estimates in different phases to the same baseline coordinate for comparison.  Figure 12 illustrates the Jacobian and its rank (CDF) distribution on a transverse slice defined in baseTLC coordinate. The left column shows the intensity pattern over four different phases; the middle column shows the corresponding Jacobian maps in each phase; and the right column shows the corresponding rank maps of Jacobian estimates. 

**Figure 13:**
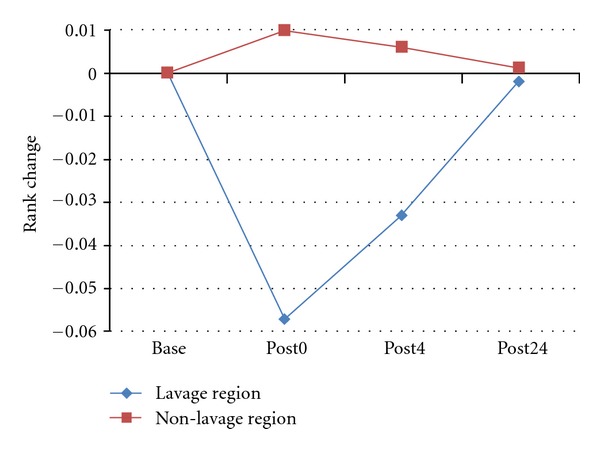
Observance of regional function change over time by tracking the rank change of Jacobian estimates over four phases within lavage region and non-lavage region.

As shown in Figure 12(d), the region with unretrieved BAL (lavage region) has much higher CT intensity than other regions. This intensity difference enables us to segment the lavage region manually. The regional function change over time can be observed by tracking the rank change of Jacobian estimates over four phases within lavage region and non-lavage region, as shown in Figure 13. 

## 4. Discussion

 We have described a registration-based method to study the progress of regional BAL resolution and lung function change. Both inter-phase and intra-phase registration achieved good accuracy by visual inspection as shown in Figure 6, and by tracking landmark movement. The mean landmark (airway branch points) tracking error across six subjects is 0.70 ± 0.34 mm for inter-phase registration, and 0.86 ± 0.50 mm for intra-phase registration. These errors are within subvoxel range, which indicates that the registrations results were able to describe lung deformations within the same phase and between different phases with tolerable errors.

Inter-phase registration was used to track tissue volume change in any arbitrary-shaped lung regions through all different phases. At the whole lung level, the bronchoscopist-reported unretrieved BAL *V*_BAL_ and quantitative-assessed tissue volume changes from baseline to post0 phase TVC_1_ are listed in Table 2 for each subject. We noticed that TVC_1_ was always lower than *V*_BAL_, which may due to the fluid resolution during the time interval between lavage and the first post-lavage scan (within 30 minutes). The linear regression analysis demonstrated good correlation between bronchoscopist-reported *V*_BAL_ and quantitative-assessed TVC_1_, as shown in Figure 9(a). The correlation coefficient is *R* ^2^ = 0.81 with a slope of 0.83. After removing data from subject 2 which had inaccurate unretrieved BAL measurement due to cough, the correlation coefficient increased significantly to *R* ^2^ = 0.94 with a slope of 0.98, as shown in Figure 9(b). This analysis shows that the quantitative-assessed tissue volume change is highly correlated with bronchoscopist-measured data, which demonstrates the method we used to track tissue volume change through inter-phase registration is meaningful in the global sense.

With inter-phase registrations, we were also able to track the tissue volume change through the four phases. As shown in Table 3, the mean tissue volume change ratio to baseline state is 14% ± 5.41 at post0 phase, then it drops to 7% ± 4.59 at post4 phase, and finally decreased to 1% ± 2.64 at post24 phase. This indicates that the fluid is continuously resolved during the 24 hours after lavage. After 24 hours, most lavage fluid is absorbed and the lung tissue volumes return to their baseline state.

Besides assessing tissue volume change at the global level, we were able to track the tissue volume change within subregions defined on baseline data sets with inter-phase registration. For instance, lobar segmentation on baseline images was used to track the tissue volume change in each lobe through the four phases. As shown in Figure 10, the right middle lung and left upper lung have the largest tissue volume change after lavage. This is because lavage fluid was delivered to the two lobes. However, the other three lobes also experience tissue volume increase, which indicates the fluid was redistributed among different lobes. After 24 hours, most lavage fluid was absorbed and the tissue volume of all lobes return to baseline state.

Intra-phase registration results provides local lung function measurement assessed by Jacobian within each phase. Dividing the whole lung region into 30 slabs from dorsal to ventral along lung height, or from apex to base along lung length, the mean Jacobian was tracked in each slab region across four phases with inter-registration results. As shown in Figure 11, the mean Jacobian in each slab was high at baseline, then dropped dramatically at post0 phase, and increased at post4, and finally returns to the baseline state at post24 phase. This is because with fluid in the lung, all subjects except subject 6 have smaller inspiration effort immediately after lavage, as shown in Figure 1. As the effects of the fluid resolves, the lungs return to normal state and have similar breathing efforts with baseline state. As a result, the lung function recovers with the same inspiration efforts, shown as the overlap between blue line (baseline) and purple line (post24 phase) in Figure 11. We also noticed that there were gradients for each mean Jacobian curve from dorsal to ventral along lung height, and from apex to base along lung length. With different lung volume change, regions with larger lung function (dorsal lung and base lung) were affected more than other regions.

Figure 12 shows the local Jacobian map on a transverse slice. After warping all post-lavage images to baseline, the change on the same slice was tracked during the 24 hours. Fluid delivery and resolving is clearly seen in the intensity images. Jacobian maps show that the function decreases in post0 and post4 phases, and recovers in post24 phase. The cumulative density function (CDF) of Jacobian gives us a rank information of Jacobian, which eliminated the affect of different breathing efforts. It shows that at post0 and post4 phases, the rank for non-lavage region was almost unchanged, but the lavage region was significantly decreased. Also, after 24 hours, the rank for each region recovered. This pattern of rank change is also reflected in Figure 13. The lavage regions has around 6% function rank decrease immediately after lavage, while non-lavage regions have a little increase in contrast. Those rank changes disappear after 24 hours.

The results of this work agree with the previous study in [3–6]. References [3–5] showed that the fluid was absorbed gradually within 24 hours and [6] demonstrated the lung mechanics change after BAL. Most of those studies are at the global level, or depends on the region of interest segmentations provided for each dataset. Our work used image registration to generate dense displacement field between images and was able to track the fluid content change within any given region over time quantitatively. Lung function changes were also analyzed from registration resulting transformations.

## 5. Conclusions

 This paper described a method to study fluid resolution progress and lung function change regionally using image registration techniques. Results were presented to show that the registration is able to describe lung motion within the same phase and between different phases. Inter-phase registrations enabled us to track tissue volume change in any arbitrary-shaped lung regions through all different phases. Meanwhile, intra-phase registrations provided the local lung function map within the same phase. Combining inter-phase and intra-phase registrations, lung function change was tracked through different phases at a local region level. This method can also be applied to track disease progression and help in radiotherapy design.

## Figures and Tables

**Table 1:** Eight CT scans acquired from four phases for each subject.

	Baseline	0 hours after BAL	4 hours after BAL	24 hours after BAL
FRC scans	baseFRC	post0FRC	post4FRC	post24FRC
TLC scans	baseTLC	post0TLC	post4TLC	post24TLC

**Table 2:** Statistics of *V*_BAL_ and TVC_1_ for each subject.

Subjects	*V*_BAL_ (mL)	TVC_1_ (mL)
1	147	125.48
2	160	107.80
3	110	106.16
4	151	130.00
5	150	123.37
6	68	42.03

**Table 3:** Statistics of whole lung tissue volume change ratio TVCR_1_, TVCR_2_, and TVCR_3_ for each subject and averaged over six subjects.

Subjects	TVCR_1_	TVCR_2_	TVCR_3_
1	14%	12%	5%
2	15%	4%	1%
3	15%	3%	0%
4	22%	13%	0%
5	14%	8%	1%
6	5%	3%	2%

Avg	14% ± 5.41	7% ± 4.59	2% ± 1.94
